# Risk assessment model for international construction projects considering risk interdependence using the DEMATEL method

**DOI:** 10.1371/journal.pone.0265972

**Published:** 2022-05-20

**Authors:** Fengfeng Zhu, Hao Hu, Feng Xu

**Affiliations:** School of Naval Architecture, Ocean and Civil Engineering and State Key Laboratory of Ocean Engineering, Shanghai Jiao Tong University, Shanghai, China; Gonbad Kavous University, ISLAMIC REPUBLIC OF IRAN

## Abstract

Given the complexity of international construction projects (ICP), risk management difficulties commonly cause cost overruns. This paper analyzes the problems of risk interdependence and subjective ratings in the application of the traditional risk assessment model in ICP. To solve the above problems, this paper proposes a risk assessment model for ICP that considers risk interdependence and obtains references from similar projects. The model applies the Decision-Making Trial and Evaluation Laboratory (DEMATEL) to determine the risk interdependence and its contribution to the overall project risk. Moreover, this model recalls the risks, probabilities, impacts, and risk events records of similar historical projects as the necessary inputs, thereby addressing the issue of subjectivity. An integrated framework is provided to identify, analyze, and prioritize ICP risks to incorporate risk interdependence into the risk management process. Finally, this paper demonstrates and validates the proposed model through a real project. The proposed model is useful for international construction companies to support project selection and bidding decisions in the early stage of ICP.

## Introduction

International construction projects (ICP) are more complex than domestic construction projects because of the transnational participants, diverse currencies, different cultures, unfamiliar standards, and unpredictable nature of disputes in different language versions of the contract. The complexity of ICP leads to risk management difficulties [1] that may cause delays [2] and cost overruns [3]. Identifying, analyzing, and prioritizing risks in the early proposal stage of ICP can construct appropriate risk assessment and conduct project in its acceptable way [4], thereby deserving scholarly focus.

Previous studies have proposed a comprehensive checklist of risk factors, quantified the probability and impact of the risks using fuzzy set theory, weighed the importance of each risk by the analytic hierarchy process (AHP), and finally completed the prioritization of individual project risks or rating of overall project risk. However, these methods or techniques focus on assigning subjective ratings to individual project risks, resulting in two challenges.

The first challenge is the ignorance of risk interdependence. Previous studies mostly modeled project risk as a group of parallel risk factors [5–7] under the assumption that the risks were independent of each other. Hence, these studies concentrated on improving the quantification of risk probability and impact. Although some studies have analyzed risk manageability or predictability, they have also failed to reveal the interdependence between risks and the impact of risk interdependence on projects [8]. If risk interdependence can be considered during risk assessment, the effectiveness of risk management can be promoted to a great extent, which is very important for ICP because ICP has more risk interactions than domestic construction projects due to country-related risks.

The second challenge is subjectiveness. In practice, decision-makers inevitably rely on personal experience, intuitive judgment, and risk preference to judge the risk probability and impact. It is practicable in domestic construction projects because of the similar project environment. However, the international construction market is diverse and changeable, which increases the difficulty of identifying comparable projects based on personal experience. Occasionally, this leads to vagueness and inconsistencies during risk assessments for ICP. If the decision-maker can recall historical projects in an extensive organizational repository and conduct risk assessments based on this documented information, such a problem can be avoided.

Therefore, this paper proposes a risk assessment model for ICP that considers risk interdependence and obtains references from similar projects. This model applies the Decision-Making Trial and Evaluation Laboratory (DEMATEL) to determine the risk interdependence and its contribution to the overall project risk. Among a great variety of multiple-criteria decision-making (MCDM) methods, DEMATEL is able to visualize the complex interdependence among criteria [9]. Hence, it shows the potential to identify the critical risk of a project [10]. Moreover, the model recalls the risks, probabilities, impacts, and risk events records of similar historical projects as the necessary inputs, thereby addressing the issue of subjectivity.

The remainder of this paper is organized as follows. The “Literature review” section reviews the literature on ICP risk management, risk interdependence, and DEMATEL. The “Risk assessment model” section describes the proposed model considering risk interdependence. The “Case study” section presents a case study to demonstrate the model application and validate the effectiveness of the proposed model. Finally, the “Conclusions” section concludes the paper by summarizing the contributions and limitations of this research and providing suggestions for future research.

### Literature review

The application of MCDM methods for risk assessment in construction projects has abounded in literature, as shown in Table 1. However, most methods assume that the criteria are independent and fail to consider their interactions. Although ANP, an advanced version of the AHP, can deal with the interdependence between criteria, the assumption of equal weight for each cluster to obtain a weighted supermatrix is not reasonable in practical situations [11].

**Table 1:** Application of MCDM methods for risk assessment in construction projects.

MCDM methods	Brief introduction	References	Interdependence between criteria
Analytic Hierarchical Process (AHP)	AHP constructs a hierarchy and determines the weights of criteria by pairwise comparison separately.	Mustafa et al. [12], Dikmen and Birgonul [13], Okudan and Budayan [14], Koulinas et al. [15], Liu et al. [16], Maceika et al. [17], Zhong et al. [18], Serrano-Gomez and Ignacio [19];	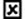
Analytic Network Process (ANP)	ANP is a generalization of AHP involving the interdependence among criteria.	Bu-Qammaz et al. [20], Valipour et al. [21], Karamoozian et. al. [22], Almeida and Oreta [23], Gashaw and Jilcha [24], Erol et al. [25];	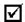
Best-Worst Method (BWM) [26]	BWM utilizes two sets of pairwise comparisons (the best criteria with the others and the worst criteria with the others) to find the optimum proportion of weights and consistency	Luo et al. [27], Wang and Jin [28], Mahmoudi et al. [29], Celik and Gul [30], Faraji et al. [31];	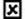
Weighted Aggregated Sum Product Assessment (WASPAS) [32]	WASPAS applies a joint criterion for determining the total importance of alternatives, giving weighted contribution of Weighted Sum Method (WSM) and Weighted Product Method (WPM) for a total evaluation	Dejus and Antucheviciene [33]; Vafaeipour et al. [34], Alvand et al. [35], Badalpur and Nurbakhsh [36];	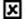
COmplex PRoportional Assessment (COPRAS)	COPRAS ranks alternatives via obtaining their significance and utility degree.	Zavadskas et al. [37], Valipour et al. [38], Valipour et al. [39], Valipour et al. [40], Ehsanifar and Hemesy [41];	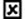
Technique for Order Performance by Similarity to Ideal Solution (TOPSIS)	TOPSIS finds the optimal alternative with the shortest distance from the positive ideal solution and the farthest distance from the negative ideal solution simultaneously.	KarimiAzari et al. [42], Taylan et al. [43], Koulinas et al. [44], Wu et al. [45], Koulinas et al. [46], Tamosaitiene et al. [47];	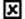
Vise Kriterijumska Optimizacija I Kompromisno Resenje (VIKOR)	VIKOR chooses the compromise solution from rank lists of distance to the ideal solution.	Zolfaghari and Mousavi [48], Gul et al. [49], Mete et al. [50], Koc and Gurgun [51];	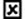
Evaluation based on Distance from Average Solution EDAS [52]	EDAS uses positive and negative distances from the average solution for appraising alternatives.	Yazdani et al. [53], Hou et al. [54], Li et al. [55]	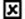

On the other hand, recent studies have indicated that risks in ICP are interdependent and such interdependence has an influence on project outcomes. They identified the major risk interdependence (also called risk paths, risk causal relations, or risk chains) by statistical techniques, such as structural equation modeling [56–60], factor analysis [61, 62], and network analysis [63]. In this case, the aforementioned MCDM methods cannot consider the risk interdependence in ICP. As a result, decision-makers still ignore risk interdependence and use subjective judgment in practice due to the lack of an integrated framework that can assign risk ratings based on historical information and assess risks considering risk interdependence.

Only a few studies have made efforts to analyze the influence of risk interdependence on project outcomes. Several authors have applied the Bayesian belief network (BBN) to assess project risk [64–66]. For example, Guan et al. [64] developed a risk assessment model for international construction projects by integrating fault tree analysis and fuzzy set theory with a Bayesian belief network; and Islam et al. [65, 66] integrated a modified Bayesian belief network model and the fuzzy group decision-making approach (FGDMA) for cost overrun risk assessment in a complex and uncertain project environment. Nevertheless, BBN is inherently acyclic and hence cannot model the loop phenomenon, namely, a causal path that leads from the initial occurrence of an event to the triggering of subsequent consequences until the initial event occurs once more. The ignorance of the loop phenomenon may lead to disasters in practice [67]. In addition, network theory and matrix tools (such as interpretive structural models and design structure matrices) are frequently used to conduct topological analyses of risk interdependence and determine the key risks [67–71]. Fang and Marle [68] applied the design structure matrix to model the risk network for decision support in project risk management. Furthermore, Fang et al. [67] conducted a topological analysis to identify key elements in the structure of interrelated risks that could potentially affect a large engineering project. However, such tools adopt "0" and "1" to indicate whether the two risks are interdependent, which may lead to the underestimation of relatively weak interdependence and overestimation of relatively strong interdependence. Moreover, some historical information from past projects is required to be input into the model for objective analysis, which is not mentioned in these studies.

Given the limitations of previous studies, this paper adopts the technique of DEMATEL to address the challenges associated with risk interdependence. DEMATEL, first developed by Gabus and Fontela [72], is a system analysis technique that uses graph theory and matrix tools to examine and solve complicated problems. Through the direct dependence matrix of the elements in the system, DEMATEL calculates the depending degree and the depended degree of each element to determine the position of each element in the system [73]. Some researchers [9, 10, 22, 74] have utilized DEMATEL to consider the interdependence between criteria. For example, Hatefi and Tamosaitiene [9] used DEMATEL to determine the interrelationships and interdependencies among risk factors, thereby extracting the network structure for implementing the fuzzy ANP method. Dehghani et al. [10] determined the critical risks associated with the process of construction using DEMATEL. Compared with previously proposed methods, DEMATEL can model the loop phenomenon and allows the description of the strength of risk interdependence. Hence, DEMATEL shows great potential in analyzing risk interdependence during a risk assessment.

Furthermore, this paper adopts the idea of case-based reasoning (CBR) to avoid subjectivity. CBR solves new problems by referring to the proven outcomes of similar situations rather than explicit formulas or predefined rules. Several researchers [1, 75–77] have demonstrated the effectiveness of CBR in recalling similar historical projects to provide a starting point for managing risk in a new project. Decision-makers can refer to the historical risk events and outcomes of similar projects to increase the reliability of decisions on current projects. However, the aforementioned studies focused on the retrieval algorithm to find the most similar project and failed to describe how to reuse the retrieved similar projects (the starting point) in the risk management process of the current project.

In summary, this paper tries to propose a risk assessment model for ICP that considers risk interdependence by DEMATEL and uses references from similar historical projects, thereby filling the previously mentioned research gap.

### Risk assessment model

**Fig 1:**
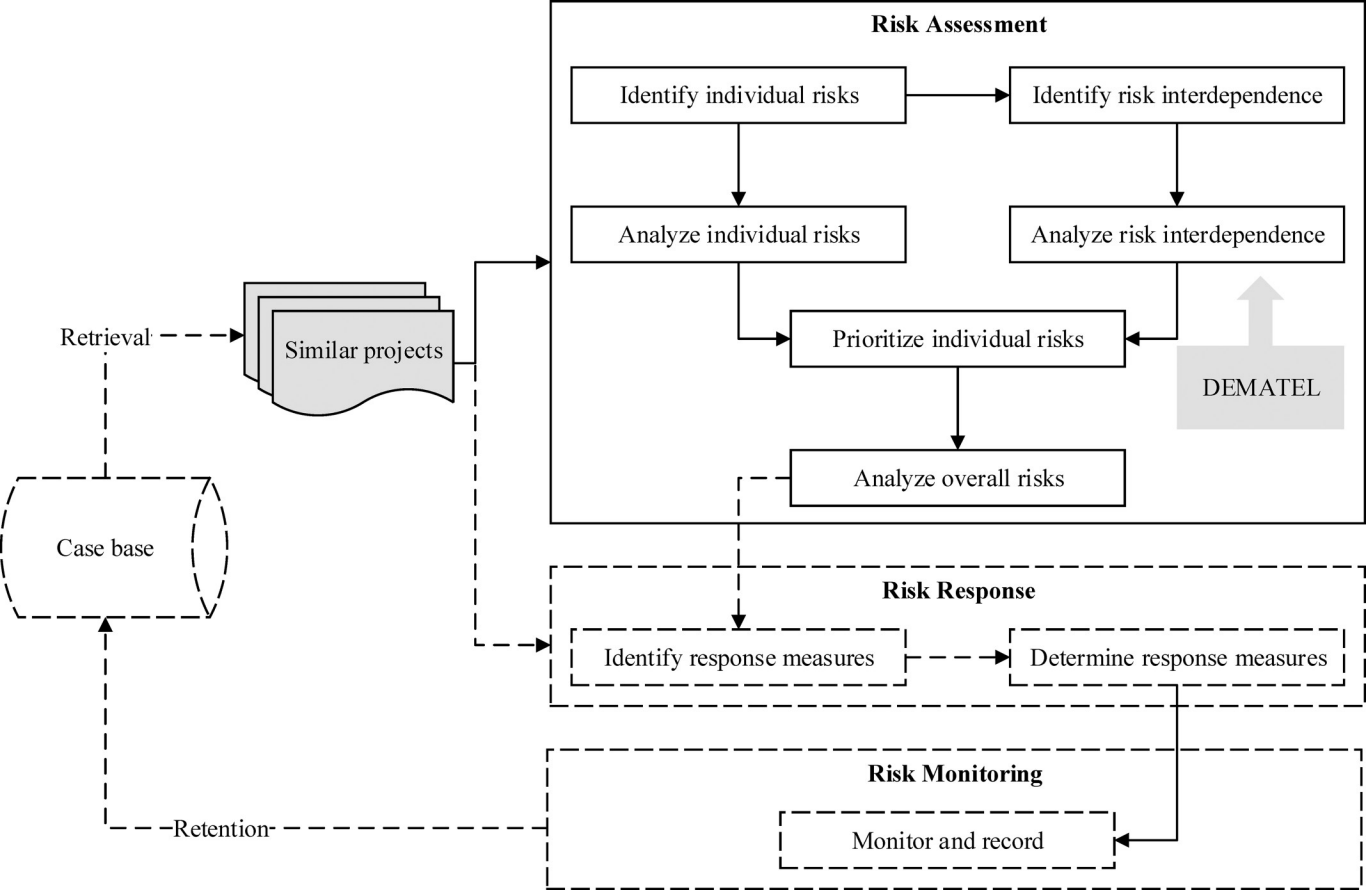
Risk assessment model for international construction projects that considers risk interdependence.

The risk assessment model for ICP that considers risk interdependence is shown in the solid line in Fig 1. The model reuses the proven outcomes of similar historical projects as the input and incorporates the result of risk interdependence analysis into risk assessment. In addition, this section proposes an integrated framework to identify, analyze, prioritize, and respond to risks in ICP, as shown by the dotted line. It is worth noting that the case base and the retrieval algorithm are not within the scope of this paper. Detailed descriptions of the case base and retrieval algorithm are provided in a previous study by the authors [1]. In brief, the previous study has constructed a case base including 102 overseas rail projects with an even distribution of data. All the projects and their risks (and risk events) were collected through post-project reviews with project managers. Besides, it proposed a retrieval algorithm based on the critical features of the project. Hence, it can deduce the most similar and relevant cases from a wide variety of historical projects.

### Identify and analyze individual project risks

As defined by PMBoK [78], each project contains individual project risks that can affect the achievement of project objectives as well as the overall project risk that arises from the combination of individual project risks and other sources of uncertainty. The identification of individual project risks requires a risk checklist first. Previous studies have proposed several classical risk breakdown structures that have been widely cited in the field of construction management. For example, Zhi [7] divided risks into four levels: nation/region, industry, company, and project. Hastak and Shaked [79] divided risks into three levels: macro (country), market, and project. Han and Diekmann [80] divided risks into political risk, economic risk, cultural/legal risk, construction risk, and other risks. El-Sayegh [81] divided risks into external risks and internal risks. Fidan et al. [82] divided risks into countries, participants, companies, and projects. In addition to the above literature, this paper extensively reviews the latest research on overseas risks to propose the risk checklist of this paper.

This paper classifies individual project risks into four levels: nation/region, trans-nation, organization, and construction, as shown in Table 2. A useful characteristic of the proposed checklist is that it clearly distinguishes the risks related to nation and trans-nation. The nation/region level risks come from nation-related factors and will have impacts on the project whether it is contracted by a foreign company or a local company. Trans-nation level risks are risks specific to foreign companies that contract construction projects across the border, such as the deterioration of diplomatic relations between the two countries. Another merit is that the proposed checklist emphasizes the health, safety, and environment (HSE) risk at the construction level, which has rarely been mentioned in previous studies. Xia et al. [83] has criticized the construction industry for not paying enough attention to environmental protection and calls on construction companies to pay more attention to HSE risk to prevent adverse public opinion regarding the project. Some countries or regions have strict penalties for environmental pollution caused by construction.

**Table 2:** Risk checklist for international construction projects.

Risk level	Risk name	Risk events
Nation/Region level	Political risk	War and conflict; Regime change; Bureaucracy; Revocation of license; Nationalization and expropriation
	Economic risk	Economic restructuring; Inflation; Interest rate change; Tax rate change
	Legal risk	Immature local laws; Complex procedures of planning approval
	Labor risk	Strong labor union; Lack of labor; Strike
	Market risk	Lack of materials; Lack of equipment; Lack of production factors; Poor logistics infrastructure
	Social risk	Bribery and corruption; Poor public security; Negative media reports
	Public health risk	Epidemic diseases; Lack of clean water; Poor environment; Poor medical infrastructure
Trans-nation level	Multilateral policy risk	Deterioration of diplomatic relations; Trade protectionism; Lack of support for foreign investment; Lack of trade agreements; Sovereign debt restructuring
	Capital liquidity risk	Exchange rate change; Restricted exchange; Mandatory agency; Mandatory contributions; Double taxation
	Material transportation risk	Import and export restrictions; Rising cost of cross-border transportation; delay in cross-border transportation; Cumbersome customs clearance procedures; Rising tariff
	Expatriate risk	Bilateral policy on expatriate; Local labor quotas; Work visa
	Standard difference risk	Different legal systems; Transnational claims and litigation; Different environmental impact assessment (EIA) requirements; Different design standards; Different construction standards; Other local special standards
	Cultural difference risk	Xenophobia; Religious differences; Language barrier; Cultural incompatibility
Organization level	Owner risk	Strong owners; Delay in payment; Delay in site delivery; Nominated subcontractors; Engineering changes; Unilateral breach of contract; Bankruptcy
	Partner risk	Poor performance; Poor communication; Unilateral breach of contract
	Contract risk	Nonstandard contract; Different language versions; Vagueness of contract condition
	Internal risk	Lack of key technologies; Lack of technical personnel; Lack of experience; Poor management ability; Multi-project; Poor cost estimation; High financing cost; Cash flow fracture
Construction level	Health, safety, and environment (HSE) risk	Safety accident; Health damage; Environmental pollution; Security incidents like kidnapping, hijacking, etc.
	Natural risk	Unforeseen geological conditions; Unforeseen climatic conditions
	Design risk	Design defects; Poor constructability; Delay in design
	Technical risk	Unfamiliar technologies; Poor construction organization design; Poor construction quality
	Human risk	Incompetence; Human error; Low efficiency; Discontent within the staff
	Material risk	Unfamiliar materials or equipment; Delay in materials or equipment; Poor quality of materials or equipment

The model assumes that the risks that occurred in similar historical projects are likely to reoccur in the current project, which is also the core idea of CBR. Therefore, the risks of the current project can be identified as the union of the risks of all similar projects. Suppose there are *E* similar projects that are sorted as {*SP*_1_, *SP*_2_,⋯,*SP*_*E*_} according to the Global Similarity Score (GSS). Suppose that *SP*_***i***_(***IR***) refers to the set of risks of the *i*^*th*^ similar project; then, *CP*(***IR***), which refers to the set of risks of the current project, can be identified as shown in Eq (1).


$$CP(IR)={SP}_{1}(IR)\cup {SP}_{2}(IR)\cup \ldots \cup {SP}_{E}(IR)$$ (1)

Next, the model analyzes the occurrence probability and the direct impact of the identified risks. In this step, the indirect impact of an individual project risk on the project outcomes by causing changes in other risks is not considered. The occurrence probability and direct impact of the risks that occurred in historical projects were collected from their risk registers. The direct impact is expressed as the ratio of cost loss and budget at completion. This paper adopts a five-point logarithmic scale to convert qualitative scales to quantitative measures, as shown in Eqs (2) and (3). In practice, the risks that cause huge losses to ICP are often small probability events. Similarly, the direct impacts of most risks are often small compared with the project scale. In this case, a logarithmic scale allows uneven distribution of probability and impact and devotes more space to small values, thereby expending the difference in risk ratings. In Eqs (2) and (3), *PS*_*ij*_ and *IS*_*ij*_ refer to the qualitative scale of probability and impact of the *j*^*th*^ risk in the *i*^*th*^ similar project, respectively, *P*_*ij*_ and *I*_*ij*_ refer to their quantitative values, respectively, and α and β are constants to adjust the range of logarithmic scales. In this paper, *α*_1_ = 2, *β*_1_ = 3, *α*_2_ = 1, *β*_2_ = 6. For ease of use, Table 3 clarifies the logarithmic value and range.


$$P_{ij}=\left\{ \begin{array}{ll} \;0, & {PS}_{ij}=0\\ \alpha_{1}\times 10^{\left(\frac{-\beta_{1}}{{PS}_{ij}}\right)}, & {PS}_{ij}\in \{1,2,3,4,5\} \end{array}\right.$$ (2)

$$I_{ij}=\{\begin{array}{ll} \;0, & IS_{ij}=0\\ \alpha_{2}\times 10^{\left(\frac{-\beta_{2}}{IS_{ij}}\right)}, & IS_{ij}\in \{1,2,3,4,5\} \end{array}$$ (3)

**Table 3:** Five-point logarithmic value and range.

Linguistic variables	Qualitative scales	Quantitative probability	Lower bound	Higher bound	Quantitative impact	Lower bound	Higher bound
Very low	1	0.2%	0	2%	0.0001%	0	0.01%
Low	2	6.32%	2%	12%	0.1%	0.01%	0.4%
Medium	3	20%	12%	28%	1%	0.4%	1.9%
High	4	35.57%	28%	43%	3.16%	1.9%	4.6%
Very high	5	50.24%	43%	100%	6.3%	4.6%	-

To apply historical projects, Okudan et al. [75] suggested determining the risk probability by counting its occurrence within the retrieved similar projects. However, this paper suggests weighing the importance of the retrieved similar projects according to their GSS. In addition, the challenge of unreporting and underreporting risks in construction industries needs to be considered. Otherwise, the unreported risks may lead to an underestimation of the risk probability and impact. Hence, this paper tries to propose a new weighting method to solve the problems. Suppose that *P*_*ij*_ and *I*_*ij*_ refer to the probability and impact of the *j*^*th*^ risk in the *i*^*th*^ similar project, respectively; then, *P*(*IR*_*j*_) and *I*(*IR*_*j*_) that refer to the probability and impact of the *j*^*th*^ risk in the current project can be defined as shown in Eqs (4), (5) and (6).


$$W_{ij}=\left\{ \begin{array}{ll} \;0, & P_{ij}=0\;\mathrm{and}\;I_{ij}=0\\ \frac{2^{E-i}}{\sum_{i=1}^{E}2^{i}}, & P_{ij}\neq 0\;\mathrm{or}\;I_{ij}\neq 0 \end{array}\right.$$ (4)

$$P({IR}_{j})=\frac{\sum_{i=1}^{E}W_{ij}\times P_{ij}}{\sum_{i=1}^{E}W_{ij}}$$ (5)

$$I({IR}_{j})=\frac{\sum_{i=1}^{E}W_{ij}\times I_{ij}}{\sum_{i=1}^{E}W_{ij}}$$ (6)

Finally, the expected loss (*EL*) of the *j*^*th*^ risk can be defined as the product of occurrence probability and direct impact, as shown in Eq (7).


$${EL}_{j}=P({IR}_{j})\times I({IR}_{j})$$ (7)

### Identify and analyze risk interdependence

The index of expected loss only analyzes the direct impact of an individual project risk on the project outcome. It is also necessary to analyze its indirect impact by causing changes in other risks. Compared with previous DEMATEL studies that focused on analyzing the relations between various elements, this paper introduces the cost overrun as the overall project risk into the direct risk dependence matrix to analyze the impact of a risk on the final result of the project by causing changes in other risks. The main steps are as follows.

The first step is to establish the direct risk dependence matrix (***R***). Suppose that there are *T-1* identified risks. If we consider the overall project risk as another separate element in the system, we can build a matrix of risk dependence with *T* rows and *T* columns. The element *r*_*mn*_ in the *m*^*th*^ row and the *n*^*th*^ column is determined by counting the occurrence of *n*^*th*^ risk caused by *m*^*th*^ risk in similar projects, as shown in Eq (8). The last column represents the occurrence that the corresponding risk directly led to cost overrun in similar projects. For example, if there are two records that one particular risk (or risk event) has caused another risk (or risk event) in the retrieved similar projects, the element in the corresponding row and column can be determined as “2”. Such records in similar projects were collected in advance and retained in the case base. The values in the last row that represents the impact of cost overrun on the corresponding risk are all 0 because cost overrun is considered the project outcome.


$$r_{mn}=CountIf(\langle {IR}_{m}\to {IR}_{n}\rangle \in {SP}_{i})\;i\in \{1,2,\ldots ,E\}$$ (8)

The second step is to calculate the normalized matrix (***N***). Suppose *R*_*mn*_ is the normalized value of *r*_*mn*_; then, it can be calculated by Eq (9).


$$R_{mn}=\frac{r_{mn}}{\underset{1\leq m\leq S}{\max}\left(\sum_{n=1}^{s}r_{mn}\right)}$$ (9)

Next, develop the risk interdependence matrix (***D***), as shown in Eq (10), where ***I*** is the identity matrix. Element *D*_*mn*_ refers to the summation of the interdependence of all paths from the *m*^*th*^ risk to the *n*^*th*^ risk.


$$D=N\times {\left(I-N\right)}^{-1}$$ (10)

Finally, this paper refers to the last column to assign the value of interdependence contribution (*IC*), which is defined as the impact of a risk on the cost overrun through its risk interdependence, and it can be understood as the added impact of a risk on cost overrun through risk interdependence under the same expected loss. Moreover, DEMATEL further calculates the depending degree, the depended degree, the centrality degree, and the cause degree. The depending degree (*D*) indicates the impact of corresponding individual project risk on other individual project risks, and it corresponds to the sum of each row except the last column. The depended degree ($\bar{D}$) indicates the impact of other individual project risks on the corresponding individual project risk, and it corresponds to the sum of each column except the last row. The centrality degree indicates the importance of the corresponding risk in the risk network, and it corresponds to the sum of its depending and depended degrees. The cause degree indicates the position of the corresponding risk in the risk network, and it corresponds to the difference between the depending and depended degrees. If the cause degree is greater than 0, then it is called a cause risk; otherwise, it is a result risk.

### Prioritize individual project risks and analyze overall project risk

The key risks are determined based on both the risk interdependence and expected loss. This paper defines the significance index (*SI*) by Eq (11). Suppose that *SI*_*j*_, *EL*_*j*_, and *IC*_*j*_ refer to the significance index, expected loss, and interdependence contribution of the *j*^*th*^ risk, respectively; then,

$${SI}_{j}={EL}_{j}\times {IC}_{j}$$ (11)

The overall project risk can be described as the variation range of some project performance, such as cost and duration. As it increases, the probability of achieving the overall project objectives decreases. For each OCP, this paper adds the value of the significance index to quantify the overall project risk exposure (RE), as shown in Eq (12). *RE* is an indicator of the relative risk magnitude of a project when compared with others.


$$RE=\sum_{1}^{T-1}{SI}_{j}$$ (12)

### Identify and determine the response measures

Risk response refers to implementing a set of response measures (or actions) to reduce the adverse impact of the identified risks on project objectives. This paper attempts to formulate the response measures for the current project by citing and improving the response measures from similar historical projects.

Consistent with risk identification, the response measures are also identified as the combination of response measures of similar historical projects. These measures would usually be practical and effective since they have been previously implemented before. In this way, decision-makers need not develop response measures from scratch. Moreover, if necessary, decision-makers should improve the identified measures to better adapt to any unique situation of the current project according to their knowledge and experience.

Finally, decision-makers should monitor the occurrence of risks during the life cycle of the OCP and report all the information on the risk management activities and project outcomes for future use.

### Case study

This section conducts a case study to demonstrate the application of the proposed model and validate its effectiveness in considering risk interdependence.

The Kumasi-Bechem railway is a railway construction project located in the Republic of Ghana that has been contracted by a leading Chinese construction company. The contract amount of the project is 500 million dollars, the construction period is 24 months, the payment type is a lump sum contract, and the delivery system is engineering procurement construction (EPC). Although the company has not implemented construction projects in Ghana before, it has extensive construction experience in other countries and regions. Therefore, this company has constructed a case base including 102 international railway construction projects.

Based on the above conditions, this paper retrieves similar historical projects in the case base by selecting input variables and collecting relevant data, as described in a previous study [1]. Four historical projects have GSSs higher than 85%. Table 4 shows these projects according to the GSS in descending order, as well as the probability (P) and impact (I) of the risks that occurred. According to Eq (1), the individual project risks of the current project can be identified as political risk, economic risk, legal risk, labor risk, market risk, social risk, public health risk, multilateral policy risk, capital liquidity risk, expatriate risk, standard difference risk, cultural difference risk, owner risk, contract risk, internal risk, HSE risk, natural risk, technical risk, human risk, and material risk.

**Table 4:** Probability and impact of the risks that occurred in similar projects.

Risk Name	Project 1		Project 2		Project 3		Project 4	
	(Under Construction)		(Completion)		(Under Construction)		(Under Construction)	
	P	I	P	I	P	I	P	I
Political risk	5	4	0	0	3	5	3	3
Economic risk	5	4	0	0	0	0	0	0
Legal risk	4	2	0	0	0	0	3	4
Labor risk	3	1	3	1	0	0	0	0
Market risk	0	0	0	0	0	0	3	2
Social risk	5	1	0	0	5	2	5	1
Public health risk	2	5	0	0	2	3	3	3
Multilateral policy risk	0	0	0	0	2	5	0	0
Capital liquidity risk	5	4	5	4	0	0	5	3
Material transportation risk	0	0	0	0	0	0	0	0
Expatriate risk	4	2	0	0	0	0	0	0
Standard difference risk	0	0	4	2	0	0	0	0
Cultural difference risk	0	0	4	3	4	1	0	0
Owner risk	4	3	5	5	4	2	0	0
Partner risk	0	0	0	0	0	0	0	0
Contract risk	0	0	5	3	0	0	0	0
Internal risk	3	4	5	5	3	2	0	0
HSE risk	3	1	3	1	0	0	5	2
Natural risk	5	2	4	2	5	3	0	0
Design risk	0	0	0	0	0	0	0	0
Technical risk	0	0	5	5	3	1	0	0
Human risk	5	1	3	2	0	0	0	0
Material risk	5	1	0	0	0	0	0	0

Eqs (2)–(6) are applied to calculate the occurrence probability and direct impact of the identified risks. Taking political risk as an example, the qualitative scale of its probability is (5, 0, 3, 3) in similar projects. Hence, its quantitative probabilities are 50.23%, 0, 20%, and 20% and the corresponding weights are 53.33%, 0, 13.33%, and 6.67%, respectively. Then, we can calculate the probability of political risk according to Eq (5) and finally obtain a value of 41.99% and a scale of 4. The second similar project (Project 2) was excluded through the weighting method to avoid underestimation of the probability because Project 2 did not report political risk. Finally, the analysis results of individual project risks are shown in Table 5.

**Table 5:** Analysis results of individual project risks for the target project.

No	Risk Name	Probability	Probability Scale	Impact	Impact Scale	Expected Loss
1	Political risk	41.99%	4	3.54%	4	1.48551%
2	Economic risk	50.24%	5	3.16%	4	1.58866%
3	Legal risk	33.84%	4	0.44%	3	0.14902%
4	Labor risk	20.00%	3	0.00%	1	0.00002%
5	Market risk	20.00%	3	0.10%	2	0.02000%
6	Social risk	50.24%	5	0.02%	2	0.00917%
7	Public health risk	7.57%	2	4.86%	5	0.36794%
8	Multilateral policy risk	6.32%	2	6.31%	5	0.39905%
9	Capital liquidity risk	50.24%	5	3.00%	4	1.50506%
10	Expatriate risk	35.57%	4	0.10%	2	0.03557%
11	Standard difference risk	35.57%	4	0.10%	2	0.03557%
12	Cultural difference risk	35.57%	4	0.67%	3	0.23715%
13	Owner risk	39.76%	4	2.39%	4	0.94971%
14	Contract risk	50.24%	5	3.16%	4	1.58866%
15	Internal risk	28.64%	4	3.62%	4	1.03802%
16	HSE risk	26.05%	3	0.02%	2	0.00523%
17	Natural risk	46.05%	5	0.23%	2	0.10523%
18	Technical risk	40.16%	4	4.21%	4	1.68955%
19	Human risk	40.16%	4	0.03%	2	0.01341%
20	Material risk	50.24%	5	0.00%	1	0.00005%

**Fig 2:**
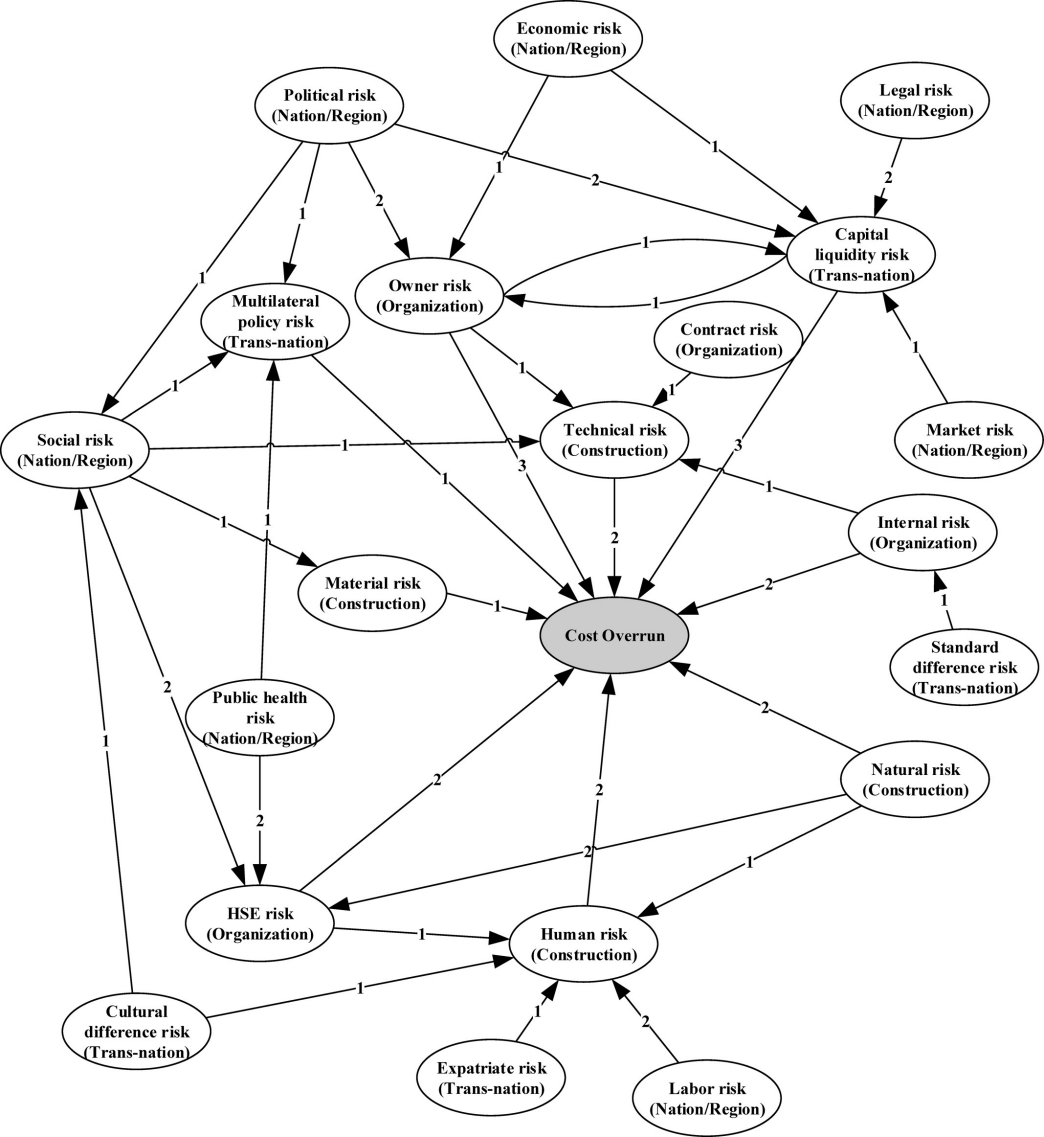
Diagram of the initial risk network for the target case.

Then, this paper counts the occurrence of risk dependence in the four similar projects. For each project, the case base retains the occurred risk events and the risk network that reflects the causal relations among these risk events. Such data was collected through post-project reviews with project managers. The risk networks of the four similar projects can be seen in S1–S4 Figs. This paper obtains the risk network for the target project by adding the risk networks of the four similar projects together, as shown in Fig 2. The arrows in the figure refer to the risk propagation directions. The numbers on the arrows refer to the number of occurrences. For example, “political risk → capital liquidity risk” indicates that the changes in political risk led to the changes in capital liquidity risk, thereby causing cost loss of the project. The number “2” indicates that 2 relevant records are obtained in these four projects.

Based on the initial risk network, this paper establishes the direct risk dependence matrix (***R***). and then calculated the normalized matrix (***N***) and the risk interdependence matrix (***D***) according to Eqs (9) and (10). The calculation process can be seen in S1 File. Hence, this paper obtains the interdependence contribution (IC), depending degree, depended degree, centrality degree, and cause degree of each risk, as shown in Table 6. According to the cause degree presented in Table 6, political risk, social risk, public health risk, and natural risk are the cause risks that generally have a significant impact on other risks. Multilateral policy risk, capital liquidity risk, owner risk, HSE risk, technical risk, human risk, and material risk are the resulting risks that are vulnerable to changes in other risks. According to the centrality degree presented in Table 5, political risk, social risk, capital liquidity risk, owner risk, HSE risk, and human risk are node risks located in the center of the risk network, and such risks interact frequently with other risks. However, expatriate risk, standard difference risk, and contract risk are relatively isolated from the other risks.

**Table 6:** Result of the risk interdependence analysis.

Risk	Interdependence Contribution	Depending Degree	Depended Degree	Centrality Degree	Cause Degree
	(*IC*)	(*D*)	($\bar{D}$)	($D+\bar{D}$)	($D-\bar{D}$)
Political risk	0.490	1.348	0	1.348	1.348
Economic risk	0.211	0.433	0	0.433	0.433
Legal risk	0.203	0.410	0	0.410	0.410
Labor risk	0.111	0.333	0	0.333	0.333
Market risk	0.102	0.205	0	0.205	0.205
Social risk	0.241	0.889	0.333	1.222	0.556
Public health risk	0.157	0.556	0	0.556	0.556
Multilateral policy risk	0.167	0	0.556	0.556	-0.556
Capital liquidity risk	0.610	0.229	1.314	1.543	-1.086
Expatriate risk	0.056	0.167	0	0.167	0.167
Standard difference risk	0.065	0.194	0	0.194	0.194
Cultural difference risk	0.096	0.481	0	0.481	0.481
Owner risk	0.657	0.371	0.886	1.257	-0.514
Contract risk	0.056	0.167	0	0.167	0.167
Internal risk	0.389	0.167	0.167	0.333	0
HSE risk	0.389	0.167	1.111	1.278	-0.944
Natural risk	0.519	0.556	0	0.556	0.556
Technical risk	0.333	0	0.898	0.898	-0.898
Human risk	0.333	0	1.185	1.185	-1.185
Material risk	0.167	0	0.222	0.222	-0.222

After analyzing the individual project risks and their interdependence, this paper calculates the significance index of each risk according to Eq (11). Table 7 presents the expected loss, significance index of each risk, and its corresponding ranks.

**Table 7:** Results for the expected loss, significance index, and corresponding ranks.

Risk name	Expected Loss	Significance Index	EL Ranking	SI Ranking	Risk events in similar projects
	(×10^−7^)	(×10^−7^)			
Political risk	148551.27	72808.46	5	2	Bureaucracy
Economic risk	158865.65	33538.3	2	6	Inflation
Legal risk	14901.81	3027.67	11	11	Complex procedures of planning approval
Labor risk	2	0.22	20	20	Many different local labor unions
Market risk	2000	203.17	15	17	Lack of materials (e.g., steel)
Social risk	917.34	220.84	17	15	Poor public security; Negative media reports
Public health risk	36793.61	5791.59	9	9	Delay due to COVID-19
Multilateral policy risk	39905.25	6650.87	8	8	Trade protectionism; Lack of support for foreign investment
Capital liquidity risk	150505.8	91736.87	4	1	Adverse change in the exchange rate
Expatriate risk	3556.56	197.59	13	18	Difficulty in applying for the work visa; Lower foreign labor quotas
Standard difference risk	3556.56	230.52	13	14	Different construction standards
Cultural difference risk	23714.54	2268.98	10	12	Religious differences (local workers do not participate in the construction during Hajj and Ramadan)
Owner risk	94971.49	62409.83	7	3	Delay in site delivery; Nominated subcontractors; Engineering changes; Delay in payment
Contract risk	158865.65	8825.87	2	7	Nonstandard contract; Different language versions
Internal risk	103801.92	40367.41	6	5	Poor cost estimation
HSE risk	523.23	203.48	18	16	Environmental pollution (e.g. wildlife habitat involved)
Natural risk	10523.31	5456.53	12	10	Extreme high temperature; Sand storm
Technical risk	168955	56318.33	1	4	Poor construction organization design;
Human risk	1341.42	447.14	16	13	Low efficiency
Material risk	5.02	0.84	19	19	Delay in materials and equipment

As shown in Table 7, after considering the contribution of risk interdependence, the risk ranking has changed. Some risks have become much less significant, such as economic risk, expatriate risk, contract risk, and technical risk. In contrast, some other risks have become much more significant, such as political risk, capital liquidity risk, owner risk, and human risk. A variety of confounding factors may underlie these results. First, the occurrence probability and direct impact were collected from the risk registers of historical projects, which are often analyzed pre-project. The risk relations of historical projects were collected from post-project reviews with project managers. Therefore, the risks that were ignored in the early stage but occurred during the project tend to become more significant, such as political risk, owner risk, and human risk. Furthermore, all the projects (including similar projects and the target project) are contracted by the same Chinese construction company. Since contract risk and economic risks have occurred and caused serious losses in a completed project (Project 2), the company inevitably would give high ratings to such risks in the risk registers of the other three similar projects under construction. Benefitting from attention and prevention, such risks have not occurred in other projects. As a result, the significance of contract risk and economic risk is decreased for the target project. In summary, according to the significance degree, the risks for the target project are ranked as follows: capital liquidity risk, political risk, owner risk, technical risk, internal risk, economic risk, contract risk, multilateral policy risk, public health risk, natural risk, legal risk, cultural difference risk, human risk, standard difference risk, social risk, HSE risk, market risk, expatriate risk, material risk, and labor risk. Notably, the above results are limited to the target projects discussed in this section.

The overall project risk exposure (*RE*) for the target project is 0.03907 according to Eq (12). Given that *RE* is a relative indicator, this paper also applied the proposed model to Project 2 for comparison. The *RE* for Project 2 is 0.03648. Hence, the risk level of the target project is slightly higher than that of Project 2. *RE* might be useful in selecting candidate projects, making bidding decisions, and formulating contingency reserves.

This paper develops a list of response measures for the target project, as shown in Table 8. These measures consist of the measures adopted in similar projects, the suggestions obtained during post-project reviews of similar projects, and the opinions from company management.

**Table 8:** List of response measures for the target project.

No	Measure Description	Classification
1	Check the terms one by one in FIDIC format to avoid ambiguity in different languages; Clarify contractor’s rights in the EPC contract.	Transfer
2	Purchase Chinese Export Credit Insurance to transfer the risk of owner bankruptcy, government prohibition or restriction of exchange, deferred payment order, war, revolution, and riot; Purchase other insurance specified in the contract.	Transfer
3	Remain strictly politically neutral in local government and parliament; Remain strictly politically neutral in different parties; Maintain communications with local chiefs.	Mitigation
4	Determine the payment option in advance; Open an offshore account in advance; Check the bilateral investment protection agreement between China and Ghana in advance.	Mitigation
5	Hire more local workers; Allocate schedule reserves to account for possible religious holidays of local workers.	Mitigation
6	Maintain communications with local labor unions; Sign a package contract to specify the minimum wage, salary increase range, etc.; Hire a local person to deal with labor disputes	Mitigation
7	Select efficient subcontractors	Mitigation
8	Maintain communications with residents; take actions to improve the community environment to avoid residents’ protests against the construction.	Mitigation
9	Pay attention to environmental protection and wildlife protection within the scope of the construction site.	Mitigation
10	Maintain communications with local media; Set up a website for information disclosure to avoid misunderstanding	Mitigation
11	Clarify the price and availability of local materials and equipment; Consider the time and cost of international transportation in the contract.	Mitigation
12	Retain the change orders from the owner in case of claims; Select an appropriate place of arbitration	Mitigation
13	Maintain communications with the Chinese medical team stationed in Ghana; Bring the necessary medications; Vaccinate Chinese employees before going abroad	Mitigation

## Conclusions

This paper analyzes the problems of risk interdependence and subjective ratings in the traditional risk assessment model for international construction projects. To solve the above problems, this paper proposes a risk assessment model for ICP that analyzes the risk interdependence through DEMATEL and obtains references from the retrieved similar projects. In addition, this paper incorporates the analyzed risk interdependence into the risk management process, thereby constructing an integrated framework to assess risks for ICP considering risk interdependence. Finally, this paper demonstrates and validates the proposed model through a real project. The contributions of this paper are as follows.

1. This paper proposes a risk checklist for ICP that classifies individual project risks into four levels: nation/region, trans-nation, organization, and construction. The clear distinction between nation level risk and trans-nation level risk is used to provide a complete description of the risks of ICP. In addition, HSE risk is listed to highlight that additional attention should be focused on environmental protection.
2. This paper provides a method of assigning weights that considers the global similarity score of historical projects and the unreporting of risks. The weighting method is helpful for updating the probability and impact of potential risks for the current project.
3. This paper describes a model for assessing project risk that considers risk interdependence. The model applies DEMATEL to analyze the interdependence contribution of risks to the project outcome and determines the significance of risks through the joint value of expected loss (direct impact) and interdependence contribution (indirect impact). Hence, it can prioritize the individual project risks and evaluate the overall project risks with consideration to risk interdependence. This step is very important for ICP because these projects tend to have more complex risk interdependence. Although some risks events did not cause cost loss directly, they may lead to the occurrence of other risk events and cause cost loss indirectly. In summary, the proposed model can support decisions related to risks in the early proposal stage of ICP.

Despite the advantages of the proposed method, there are still limitations to the conclusions. First, the proposed model relies on the support of an extensive case base that contains a wide variety of historical projects with risk events. The case base used in this paper is collected from a Chinese construction company, hence should not be directly generalized to other contractors. Instead, the contractors should construct different case bases individually following the proposed process. If there lacks such a case base, the subjective ratings from experts are required as the input to the model. Second, this paper applies the causal relations among the occurred risk events in similar projects as the input of DEMATEL. Given that such data were collected from post-project reviews with project managers, certain data deviations remained due to various reasons, such as the loss of documents, unwillingness to disclose mistakes, and risk preference of different managers. Finally, this paper emphasizes the identification and analysis of risks specific to international construction. Hence, the proposed model is useful for international construction companies to support project selection and bidding decisions in the early stage of ICP. However, it should not be directly generalized to the middle and late stages due to the differences in key points of risk management. The future work of this paper is to develop a computer-based tool to realize the proposed models. In this way, this paper could provide better decision support for international construction companies.

## Supporting information

S1 Fig Risk networks of Project 1. (TIF)

S2 Fig Risk networks of Project 2. (TIF)

S3 Fig Risk networks of Project 3. (TIF)

S4 Fig Risk networks of Project 4. (TIF)

S1 File Calculation process for DEMATEL. (XLSX)
