# Supplementary figures and images for: Risk assessment model for international construction projects considering risk interdependence using the DEMATEL method
Source: PLoS One. 2022 May 20;17(5):e0265972. doi: 10.1371/journal.pone.0265972 (PMC9122217; doi:10.1371/journal.pone.0265972)

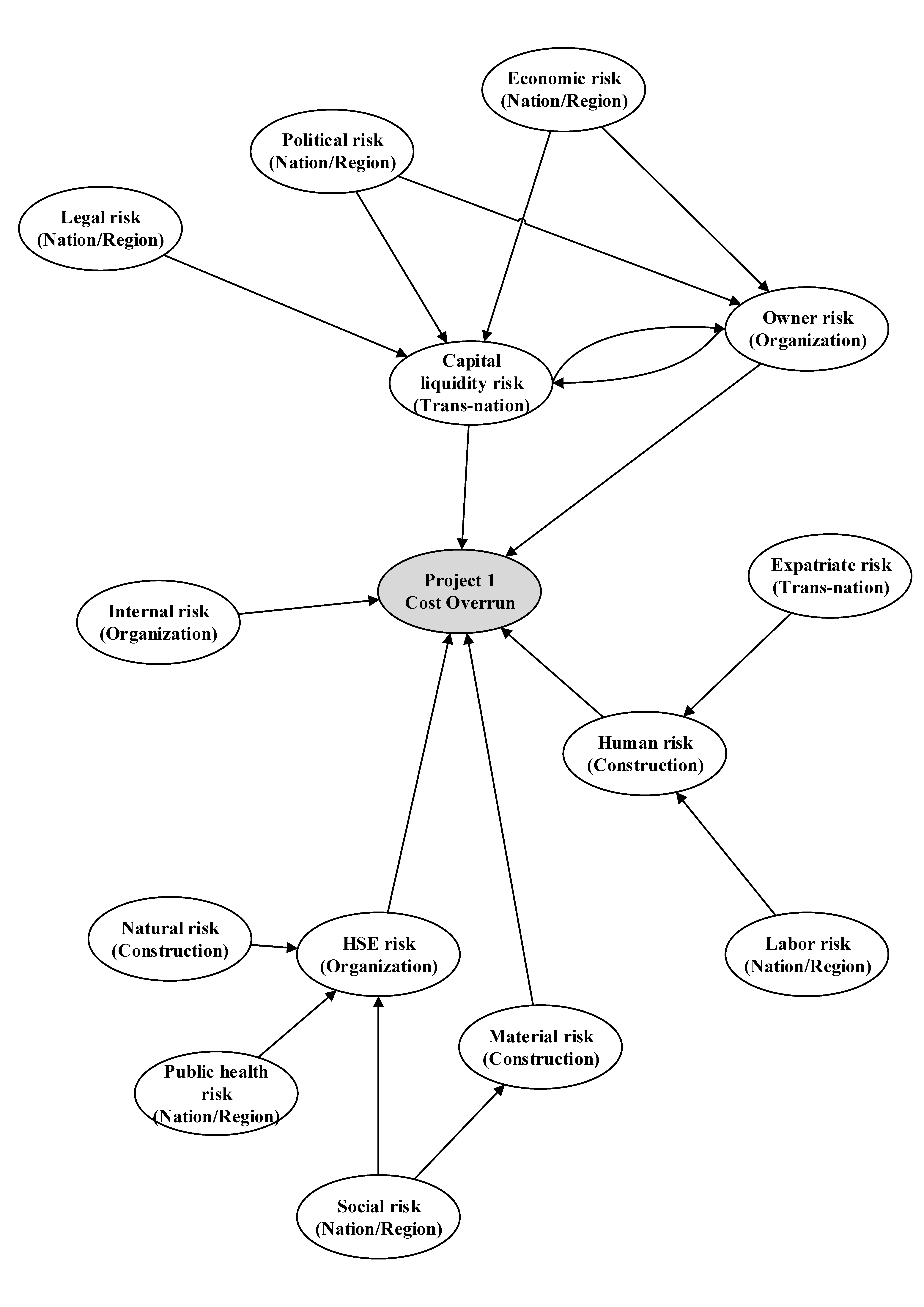

Supplement: S1 Fig — (TIF) [file pone.0265972.s001.tif]

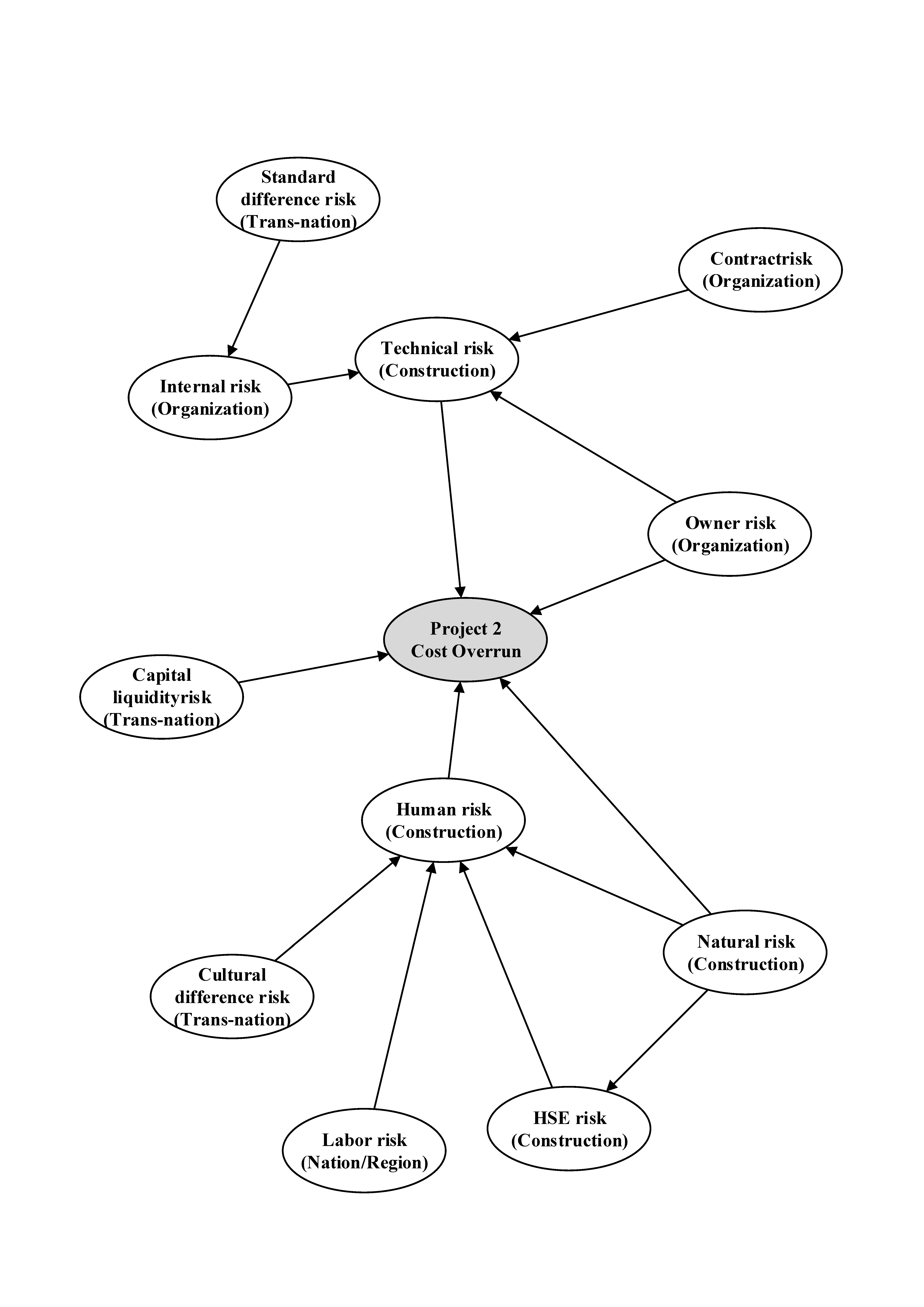

Supplement: S2 Fig — (TIF) [file pone.0265972.s002.tif]

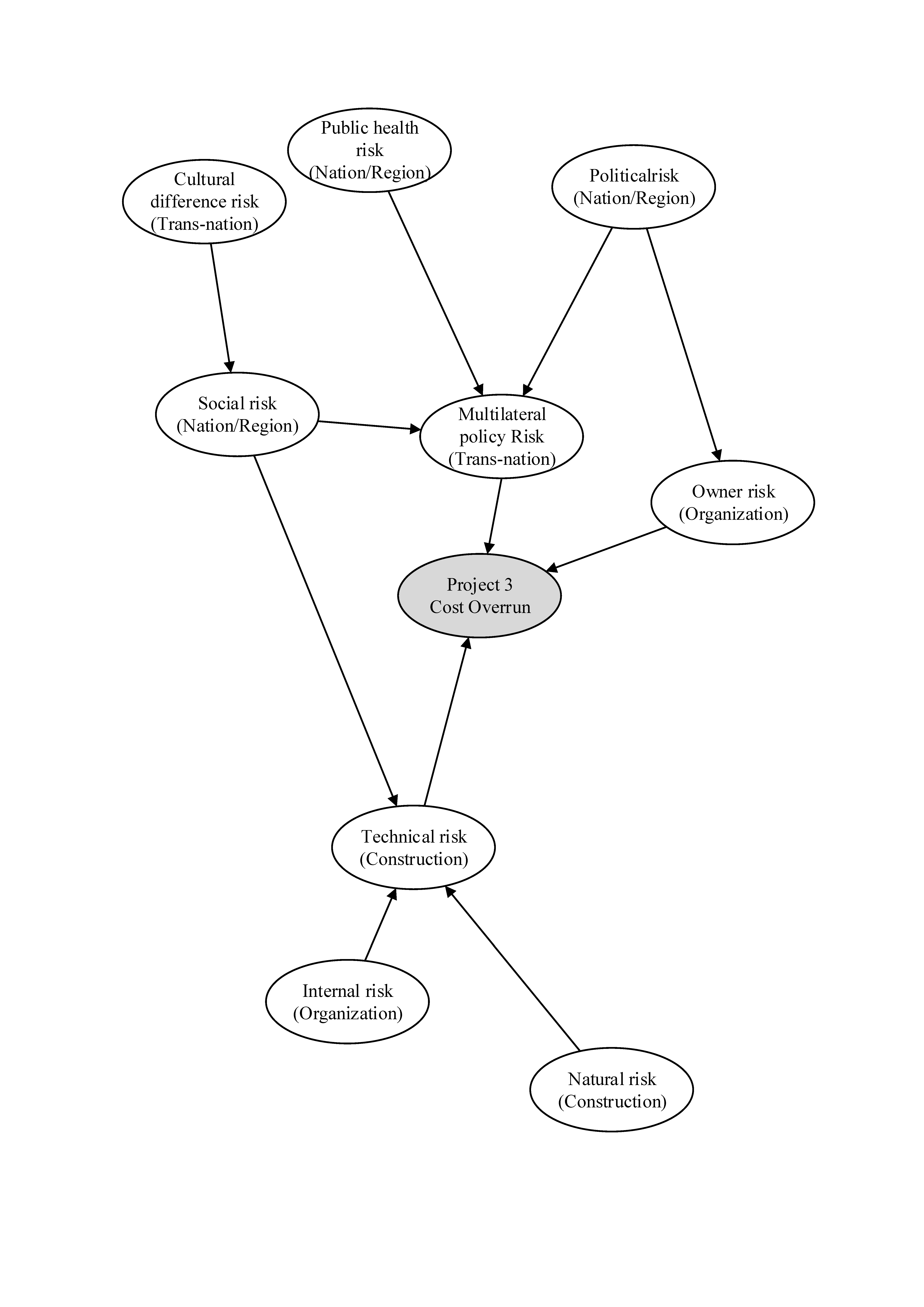

Supplement: S3 Fig — (TIF) [file pone.0265972.s003.tif]

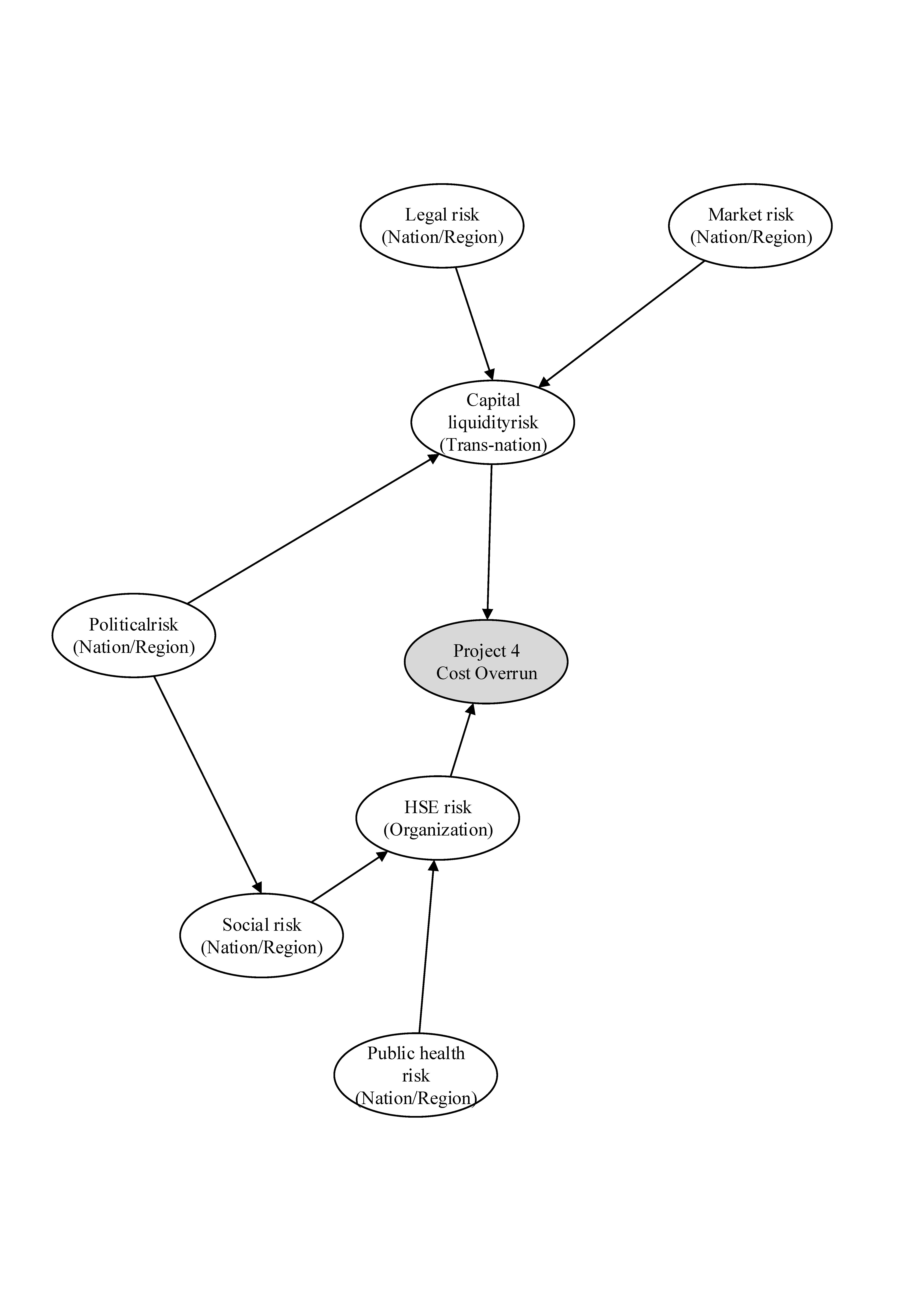

Supplement: S4 Fig — (TIF) [file pone.0265972.s004.tif]
